# First Aid for Pool Chemical Exposure: A Narrative Review

**DOI:** 10.7759/cureus.16755

**Published:** 2021-07-30

**Authors:** Jennifer A Ross, Samuel R Seitz, Peter Wernicki, Roy R Fielding, Nathan Charlton

**Affiliations:** 1 Medical Toxicology, University of Virginia School of Medicine, Charlottesville, USA; 2 Rehabilitation Science and Technology, University of Pittsburgh, Pittsburgh, USA; 3 Orthopedics, ProSports and Elite Rehab, Vero Beach, USA; 4 Kinesiology, University of North Carolina Charlotte, Charlotte, USA; 5 Emergency Medicine, University of Virginia, Charlottesville, USA

**Keywords:** first aid, pool, aquatics, chlorine, pool chemical, inhalation

## Abstract

Over 4500 visits to emergency departments related to pool chemical exposures occur annually in the United States, and it is likely that many more go unreported. Injury can occur due to sensitivity to standard chemical concentrations in pools (such as with opening one’s eyes underwater), accidental exposure to super-concentrated chemicals (such as malfunction of a pool chlorinator), or accidental inhalation of fumes while opening the storage container for chlorination tablets. Therefore, first aid care has the potential to significantly limit morbidity.

A literature review was undertaken to determine the appropriate first aid for pool chemical exposures. This literature search revealed 25 pertinent articles, of which none were systematic reviews or studies directly related to the first aid treatment of pool chemical exposures. However, five articles were included as indirect evidence, and a hand search of references revealed five additional articles for inclusion. Treatment recommendations were extrapolated from the treatment of chemical exposures from other incidents.

Symptoms of pool chemical exposure reflect irritation secondary to the caustic chemicals involved. Patients may report irritation of the skin, eyes, nose, or throat; cough; chest tightness; and difficulty breathing. Following any exposure to pool chemicals, the victim should be removed from the source, taken to an area with fresh air, and contaminated materials (e.g. clothing, contact lenses) should be removed. Irrigation of the exposed area should take place immediately with uncontaminated fresh water for at least 15 minutes. It is anticipated that an exposed person will be symptomatically improved by leaving the area of exposure, removing their contaminated clothing items, and irrigating the contaminated body parts. However, if symptoms do not resolve during that time or if symptoms are worsening, the victim should be evaluated by trained medical personnel.

## Introduction and background

It is estimated that over 4500 emergency department (ED) visits related to pool chemicals occur annually in the United States (US) per a CDC Morbidity and Mortality Weekly Report [1]. This report evaluated the incidence of pool chemical injuries in the US between 2008 and 2017 and found that most injuries were relatively minor, as 93.9% were treated and released from the ED, and no deaths were reported. Of these injuries, approximately 90% were inhalational injuries, with other injuries reported, including chemical burns, conjunctivitis, and dermatitis. Most injuries occurred at a place of residence rather than at a public facility.

Pool sanitation is important to prevent the transmission of disease, with the primary way to achieve sanitation being the use of chemicals. Chemicals used in pool treatments are typically caustic, with the primary agents being chlorine, sodium hypochlorite, or the alternative bromine, and come in the form of granules, tablets, liquids, and gasses [2]. In addition, pure hydrochloric acid or other acids may be needed to help regulate the pH under certain conditions. Chlorine reacts with pool water to form hypochlorous acid, hydrochloric acid, and small amounts of other caustic chlorinated hydrocarbons [2,3]. A similar reaction occurs with sodium hypochlorite. Both the primary compounds and the generated caustic chemicals are irritants to the skin, eyes, and mucous membranes, and when aerosolized form gases that are irritants to the respiratory tract. Injury can occur due to sensitivity to standard chemical concentrations in pools (such as with opening one’s eyes underwater), accidental exposure to super-concentrated chemicals (such as malfunction of a pool chlorinator), or accidental inhalation of fumes while opening the storage container for chlorination tablets.

While more than 4500 visits to emergency departments related to pool chemical exposure are reported each year in the United States, it is likely that many more go unreported. First aid has the potential to significantly limit morbidity. This review was undertaken as part of an American Red Cross Scientific Advisory Council scientific review in order to determine the appropriate first aid for pool chemical exposures.

## Review

Methods

A search strategy was developed with the help of a medical librarian (EA), and a single author (NC) screened articles for inclusion. An initial literature search revealed no studies or systematic reviews that directly addressed the first aid treatment of exposures to pool chemicals. With a lack of suitable literature directly related to the first aid treatment of pool chemical exposures, we conducted a hand search of book chapters and grey literature to help classify the types of chemicals that are involved in pool sanitation and maintenance to help construct a subsequent search strategy [2,3]. A subsequent systematic search was conducted using a strategy to capture literature regarding first aid for human exposures specifically to pool chemicals as well to non-pool related exposure (such as industrial exposure) to chemicals commonly used in pools (Appendix A). Systematic and narrative reviews, randomized controlled trials, and observational studies were included but limited to the English language. Due to the anticipated lack of randomized studies, case series and case reports were also included. The search was performed within MEDLINE (via the PubMed interface) from inception to August 29, 2019. A further hand search was conducted based on the bibliographies of the articles discovered in the initial search.

Because the literature identified was limited to a small number of observational studies, the results are presented as a narrative report of the literature that was found.

Results

**Figure 1:**
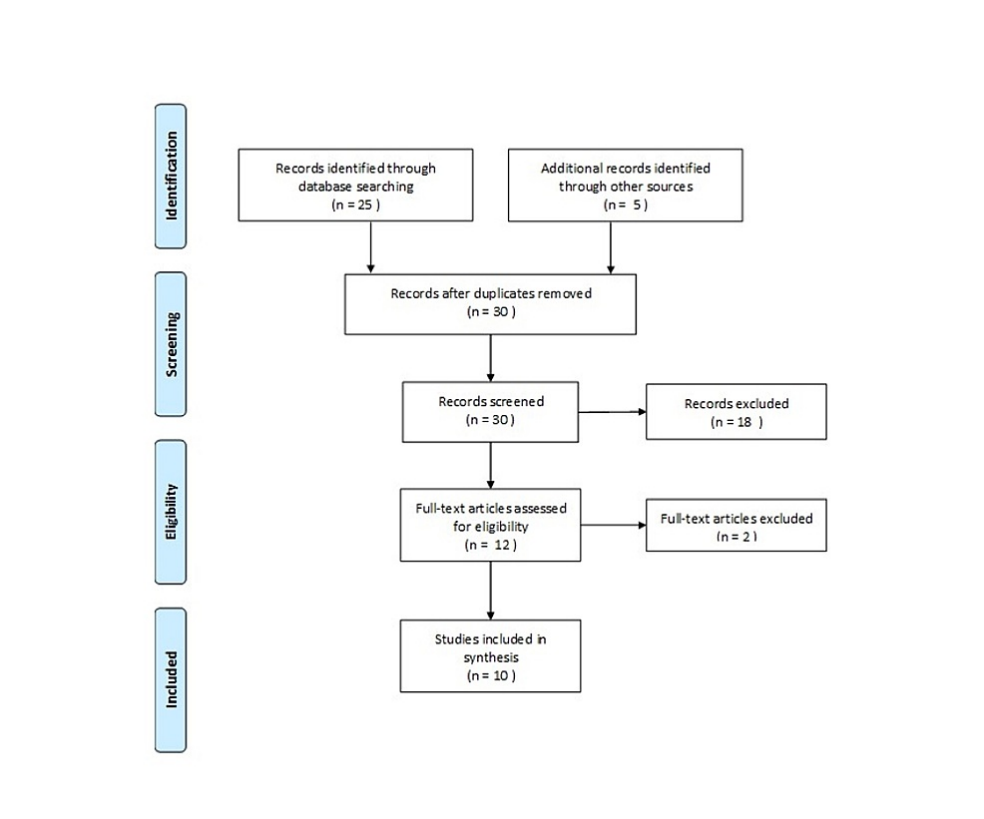
Flow Diagram of Included Articles

Twenty-five articles were found in the search (Figure 1). No studies were found that directly related to the first aid treatment of pool chemical exposure; however, five articles were included as indirect evidence from related pool and chemical exposures and background studies [1,4-7]. A hand search of references revealed five additional articles for inclusion [8-12]. All studies were observational in nature or review articles. The ten articles included in the review are summarized in Table 1.

**Table 1:** Characteristics of Included Studies

Author, Year	Type of Study	Exposure	Population	First Aid Treatment	Setting
Leonard, 1982 [11]	Case series	Dermal	35 patients presenting to a regional burn center after sustaining a chemical burn.	Immediate large volume water irrigation	Prehospital
Lawson, 1981 [4]	Case series	Inhalational	457 cases of chlorine exposure in industrial workers. Fifteen additional cases of hospitalized patients with chlorine exposure are also presented.	Supplemental oxygen	Prehospital and in hospital
Parimon, 2004 [7]	Case report	Inhalational	A case report of a 23 you male who was exposed to chlorine gas at a swimming pool.	Supplemental oxygen and beta-2 agonists	Emergency department
Ikeda, 2006 [9]	Cohort study	Ocular	Thirty-six patients (49 eyes) received immediate first aid irrigation of their eyes with tap water following alkali exposure compared with 17 patients (29 eyes) who did not receive eye irrigation in the prehospital setting.	Water irrigation vs. no irrigation	Emergency department and Ophthalmology clinic
Lehavi, 2008 [5]	Retrospective chart review	Inhalational	Review of 5 mass chlorine exposure events at swimming pools over one summer. Six events occur with a total of 81 patients.	Bronchodilators	Prehospital and Emergency department
Cevik, 2009 [8]	Case series	Inhalational	25 soldiers who were exposed to chlorine gas during cleaning activities.	Supplemental oxygen, inhaled beta-2 agonists, inhaled budesonide	Emergency department
Brent, 2013 [12]	Literature review	Dermal	Literature review that identified 69 relevant articles regarding the best decontamination technique for dermal corrosive exposures.	Water irrigation	Prehospital
Culley, 2017 [6]	Case-control study	Inhalational	One-hundred forty-six patients treated for chlorine exposure in one emergency department following a train derailment, 152 patients not exposed to chlorine served as controls.	Not discussed	Emergency department
Vanden, 2018 [1]	Retrospective chart review	Pool chemical injuries	A review of the National Electronic Injury Surveillance System database with subsequent estimates for the annual number of US emergency department visits for pool chemical injuries	Not discussed	Emergency department
Wiesner, 2019 [10]	Retrospective chart review	Ocular	One thousand four hundred ninety-five patients with 2194 chemical eye burns who received first aid eye irrigation with a variety of different substances. Six hundred sixty had irrigation done at the accident site.	Tap water or Previn® solution irrigation vs. lactated Ringer’s, normal saline, or isotonic phosphate buffer irrigation	Prehospital and Emergency department

Inhalational Exposure

Four observational studies [1,4-6], one case series [8], and one case report [7] were identified describing clinical findings and treatment of inhalational pool chemical exposure in both the prehospital setting and the emergency department. In 1981, Lawson [4] reported a retrospective chart review of 457 cases of chlorine exposure in industrial workers as well as 15 additional cases from the hospital setting. Individuals exposed were primarily treated with supplemental oxygen and supportive therapy in the prehospital setting. A retrospective chart review was published by Lehavi et al. in 2008 [5], documenting medical problems of patients with chlorine exposures from swimming pools. This article reviewed an event involving 40 children affected by chlorine exposure who experienced symptoms of dyspnea, sore throat, and eye irritation. Patients were primarily treated with oxygen prehospital and bronchodilators and steroids in the emergency department. A 2019 cross-sectional study by Vanden Esschert et al. [1], involving an estimated 13,508 emergency department visits for pool chemical injuries, reports that approximately 90% of pool chemical injuries result in inhalational exposure. In 2017, Culley et al. [6] conducted a case-control study and reported that the primary symptoms of patients with inhaled chlorine exposure included coughing and choking, dyspnea, wheezing, chest discomfort, and burning irritation of the eyes, nose, or throat. Treatment was not described. In 2009, Cevik et al. [8] reported a case series of 25 soldiers exposed to chlorine gas. The main symptoms experienced were coughing and dyspnea. These patients were treated with oxygen and inhaled beta-2 agonists, with some additionally receiving inhaled budesonide and nebulized sodium bicarbonate in the emergency department. The majority (52%) were discharged from the emergency department, and there were no deaths. In a 2004 case report by Parimon et al. [8], a patient exposed to chlorine gas from a pool chemical accident was treated with supplemental oxygen and beta-2 agonists for symptomatic relief in the emergency department.

Ocular Exposure

For ocular exposures, two observational studies [9,10] were identified from the literature search, both describing clinical signs and symptoms and first aid rendered. In 2005, Ikeda et al. [9] published an observational study of 53 patients with a caustic alkali eye injury. Patients who received first aid treatment with water irrigation between 5-60 minutes in duration had significantly shorter healing times and lower degrees of injury than those who had no initial eye irrigation (<0.05). In 2019, a retrospective chart review of 1495 patients with chemically burned eyes by Wiesner et al. [10] reported statistically significant decreased lesion severity when rinsed for 15 minutes with either tap water or Previn® solution compared with lactated Ringer’s, normal saline, or isotonic phosphate buffer (p<0.001). Tap water and Previn solution also resulted in statistically significantly less severe outcomes as defined by Reim and Roper-Hall injury grades (p<0.001). Patients in this chart review had a variety of chemical exposures, with 815 corrosive substances identified, of which 187 were cement, 178 were alkalis, and 167 were acids, among others. 

Dermal Exposure

For dermal exposures, one observational study and one literature review were identified, both discussing the initial medical management of corrosive exposures [11,12]. In 1982, Leonard et al. [11] reported an observational study of 35 patients with caustic dermal injury from a variety of agents. Those patients who received immediate irrigation within 10 minutes and with a “large volume” of water for at least 15 minutes had decreased percentage of full-thickness burns (12.5% of patients compared with 63% of patients; p<0.01) and decreased mean length of hospital stay (7.7 days compared with 20.5 days; p<0.01). Brent published a review of the literature in 2013 [12] assessing initial medical management of corrosive dermal exposures. The search found 69 relevant articles, of which five were controlled studies. After reviewing these five studies, the article concluded that due to efficacy and availability, water is the best decontaminating solution for caustic dermal exposure.

Discussion

Symptoms of caustic exposure reflect irritation of those tissues involved [1,5,7]. Exposed persons may complain of itching or burning skin as well as eye, nose, or throat pain [1,5,7]. In more severe exposures or with exposures in persons having a prior history of reactive airway disease, respiratory symptoms may include cough, chest tightness, and difficulty breathing [5-7]. A skin examination may reveal erythema or rash, and in rare instances, blistering can occur. Eye pain and redness, tearing, and difficulty opening one’s eyes may be noted on ophthalmologic examination [3].

First aid primarily consists of removal from the source and supportive care [4]. First aid providers should first ensure that the scene is safe and avoid contact with any liquid/dry chemicals, gasses, or aerosols. If there is uncertainty about the chemical involved, consider contacting emergency medical services or the poison control center. Once it is determined that the scene is safe for first aid providers, removal of the affected exposed person from the exposure to an area with fresh air is essential. Any contaminated clothing, i.e., from splashing of a liquid, should be removed if possible [4,12]. Early irrigation of both the skin and eyes is associated with improved outcomes and, therefore, should occur as rapidly as possible with a large volume of fresh water and for at least 15 minutes to treat eye or skin irritation or chemical burns [9,11,12]. While multiple irrigation solutions are available, tap water is likely to be the most abundant irrigation solution available [12]. If chemical contact with the eye has occurred and contact lenses are in place, these should be removed prior to irrigation, if possible, and not be re-used. After the person is moved to fresh air, respiratory symptoms can be treated with oxygen, if available, to relieve dyspnea and hypoxemia [7,8]. In addition, albuterol inhalers may also improve respiratory symptoms, particularly in those with a history of reactive airway disease [7,8]. However, both oxygen and albuterol may not be available to lay first aid providers, and first aid providers should always follow local regulations regarding the administration of medications. 

It is anticipated that persons with low-level exposure to pool chemicals and mild symptoms will be symptomatically improved by leaving the area of exposure, removing their contaminated clothing items, and irrigating the contaminated body parts. However, if symptoms do not resolve during that time, or if symptoms are worsening or are initially more than just mild, emergency medical services should be contacted, and the person should be evaluated by trained medical personnel. 

The limitations of this review primarily involve the paucity of literature available on pool chemical exposures and the inclusion of indirect evidence in our literature review. While a systematic search was done to identify literature on the first aid treatment of pool chemical exposures, there were no comparative studies identified that directly evaluated first aid treatment for pool chemical exposures. We attempted to identify literature pertaining to the first aid treatment of chemicals commonly used for pool disinfection in other settings, such as industrial exposures. However, it is possible that not all chemicals used in pool disinfection were identified and that not all literature pertaining to the first aid treatments for each specific chemical exposure was identified. While the inclusion of indirect literature allowed for the formulation of treatment recommendations, this narrative review strays from the formalities of a systematic review or scoping review and uses opinion to craft indirect evidence into treatments. Finally, only a single reviewer performed a screen of the articles, potentially leading to bias in the included articles.

## Conclusions

Little data is available to define the first aid treatments of pool chemical exposures. Available literature primarily comes from indirect evidence based on exposure to chemicals used in pool disinfection in other settings. In general, symptoms are caused by caustic injury and are treated similarly to other caustic exposures. In the setting of pool chemical exposures, it is important that first aid providers ensure the scene is safe, remove exposed persons from the exposure and into fresh air, and appropriately decontaminate and irrigate all exposed body areas. In cases of unknown exposures or exposed persons with worsening symptoms, first aid providers should call emergency medical services or the poison control center. As the literature suggests that first add treatment results in better outcomes, to better treat the more than 4500 known pool chemical exposures that are reported yearly in the US, more research is needed in this field.

## Appendices

**Table 2:** Appendix A Search Strategy

#		Search Statement	Results
1		First Aid/	7620
2		first aid.mp.	10257
3		first-aid.mp.	10257
4		emergency relief.mp.	147
5		emergency respon:.mp.	3512
6		initial assist:.mp.	21
7		initial care:.mp.	692
8		first assist:.mp.	428
9		immediate respond:.mp.	44
10		1 or 2 or 3 or 4 or 5 or 6 or 7 or 8 or 9	14955
11		Emergency Service, Hospital/	63489
12		Emergency Medical Services/	41049
13		emergency department:.mp.	84403
14		11 or 12 or 13	147283
15		therapy.fs.	1788534
16		treatment.ti,kf,ab.	4075438
17		15 or 16	5180628
18		14 and 17	48712
19		10 or 18	62564
20		*burns, chemical/ or *burns, inhalation/	5468
21		*Burns/	37195
22		*Eye Burns/	2067
23		toxicity.fs.	410776
24		burn:.mp.	112756
25		20 or 21 or 22 or 23 or 24	521786
26		chlorine.mp. or Chlorine/	23366
27		chloramines/ or hydrochloric acid/	9613
28		hydrochloric acid:.mp.	13514
29		exp Caustics/ or caustics.mp.	11541
30		calcium chloride.mp. or exp Calcium Chloride/	10118
31		Sodium Hypochlorite/	4529
32		bleach.mp.	2416
33		exp Swimming Pools/	1839
34		swimming pool:.mp.	2864
35		33 or 34	2864
36		26 or 27 or 28 or 29 or 30 or 31 or 32 or 33 or 34	67061
37		10 and 25 and 36	23
38		19 and 25 and 35	3
39		10 and 35	16
40		37 or 38 or 39	42
41		limit 40 to english language	25

## References

[1] Vanden Esschert KL, Haileyesus T, Tarrier AL (2019). Pool chemical injuries in public and residential settings - United States, 2008-2017, and New York, 2018. MMWR Morb Mortal Wkly Rep.

[2] Kuffner EK (2003). Athletes. Occupational, industrial, and environmental toxicology.

[3] Stolbach Stolbach, A A (2020). Tox and Hound - Lifeguard Lessions. https://emcrit.org/toxhound/lifeguard-lessons/.

[4] Lawson JJ (1981). Chlorine exposure: a challenge to the physician. Am Fam Physician.

[5] Lehavi O, Leiba A, Dahan Y (2008). Lessons learned from chlorine intoxications in swimming pools: the challenge of pediatric mass toxicological events. Prehosp Disaster Med.

[6] Culley JM, Richter J, Donevant S, Tavakoli A, Craig J, DiNardi S (2017). Validating signs and symptoms from an actual mass casualty incident to characterize an irritant gas syndrome agent (IGSA) exposure: a first step in the development of a novel IGSA triage algorithm. J Emerg Nurs.

[7] Parimon T, Kanne JP, Pierson DJ (2004). Acute inhalation injury with evidence of diffuse bronchiolitis following chlorine gas exposure at a swimming pool. Respir Care.

[8] Cevik Y, Onay M, Akmaz I, Sezigen S (2009). Mass casualties from acute inhalation of chlorine gas. South Med J.

[9] Ikeda N, Hayasaka S, Hayasaka Y, Watanabe K (2006). Alkali burns of the eye: effect of immediate copious irrigation with tap water on their severity. Ophthalmologica.

[10] Wiesner N, Dutescu RM, Uthoff D, Kottek A, Reim M, Schrage N (2019). First aid therapy for corrosive chemical eye burns: results of a 30-year longitudinal study with two different decontamination concepts. Graefes Arch Clin Exp Ophthalmol.

[11] Leonard LG, Scheulen JJ, Munster AM (1982). Chemical burns: effect of prompt first aid. J Trauma.

[12] Brent J (2013). Water-based solutions are the best decontaminating fluids for dermal corrosive exposures: a mini review. Clin Toxicol (Phila).

